# A JND-Based Pixel-Domain Algorithm and Hardware Architecture for Perceptual Image Coding

**DOI:** 10.3390/jimaging5050050

**Published:** 2019-04-26

**Authors:** Zhe Wang, Trung-Hieu Tran, Ponnanna Kelettira Muthappa, Sven Simon

**Affiliations:** Institute of Parallel and Distributed Systems, University of Stuttgart, 70569 Stuttgart, Germany

**Keywords:** just-noticeable difference (JND), luminance masking, contrast masking, texture detection, perceptual coding, JPEG-LS, downsampling, FPGA

## Abstract

This paper presents a hardware efficient pixel-domain just-noticeable difference (JND) model and its hardware architecture implemented on an FPGA. This JND model architecture is further proposed to be part of a low complexity pixel-domain perceptual image coding architecture, which is based on downsampling and predictive coding. The downsampling is performed adaptively on the input image based on regions-of-interest (ROIs) identified by measuring the downsampling distortions against the visibility thresholds given by the JND model. The coding error at any pixel location can be guaranteed to be within the corresponding JND threshold in order to obtain excellent visual quality. Experimental results show the improved accuracy of the proposed JND model in estimating visual redundancies compared with classic JND models published earlier. Compression experiments demonstrate improved rate-distortion performance and visual quality over JPEG-LS as well as reduced compressed bit rates compared with other standard codecs such as JPEG 2000 at the same peak signal-to-perceptible-noise ratio (PSPNR). FPGA synthesis results targeting a mid-range device show very moderate hardware resource requirements and over 100 Megapixel/s throughput of both the JND model and the perceptual encoder.

## 1. Introduction

Advances in sensor and display technologies have led to rapid growth in data bandwidth in high-performance imaging systems. Compression is becoming imperative for such systems to address the bandwidth issue in a cost-efficient way. Moreover, in many real-time applications, there is a growing need for a compression algorithm to meet several competing requirements such as decent coding efficiency, low complexity, low latency and high visual quality [1]. It has been realized that algorithms specifically designed to meet such requirements could be desirable [2,3,4]. Compared with off-line processing systems, the computational power and memory resources in real-time high-bandwidth systems are much more limited due to the relatively tight constraints on latency, power dissipation and cost, especially in embedded systems such as display panels for ultra high definition contents and remote monitoring cameras with high temporal and spatial resolutions.

The use of existing transform-domain codecs such as JPEG 2000 and HEVC has been limited in real-time high-bandwidth systems, since such codecs typically require storing multiple image lines or frames. Especially when the spatial resolution of the image is high, the line or frame buffers result in both expensive on-chip memories and non-negligible latency, which are disadvantages for a cost-efficient hardware implementation of the codec, e.g., on FPGAs. While JPEG-LS is considered to have created a reasonable balance between complexity and compression ratio for lossless coding, its use in lossy coding is much less widespread due to the inferior coding efficiency compared with transform-domain codecs and stripe-like artifacts in smooth image regions. It is desirable to investigate the feasibility of a lightweight and hardware-friendly pixel-domain codec with improved compression performance as well as significantly improved visual quality over that of the lossy JPEG-LS.

One possibility is to exploit the visual redundancy associated with properties of the human visual system (HVS) in the pixel domain. Features and effects of the HVS can be modeled either in the pixel domain or in the transform domain. While effects such as the Contrast Sensitivity Function (CSF) are best described in the Fourier, DCT or Wavelet domain and hence can be exploited by compression algorithms operating in these domains [5,6,7], other effects such as visual masking can be well modeled in the pixel domain [8,9]. The term visual masking is used to describe the phenomenon that a stimulus (such as an intensity difference in the pixel domain) is rendered invisible to the HVS by local image activities nearby, hence allowing a coarser quantization for the input image without impacting the visual quality. The masking effects of the HVS can be estimated by a visibility threshold measurement model, which ideally provides a threshold level under which the difference between the original signal and the target signal is invisible. Such a difference threshold is referred to as just-noticeable difference (JND) [10]. Compression algorithms like JPEG-LS operating in the pixel domain can be adapted to exploit the pixel-domain JND models, e.g., by setting the quantization step size adaptively based on the JND thresholds. One problem with such a straightforward approach, however, is that the JND thresholds must be made available to the decoder, incurring a relatively large overhead.

A classic pixel-domain JND model was proposed by Chou and Li [9]. This model serves as a basis for various further JND models proposed in research work on perceptual image/video compression, such as Yang et al.’s model [11] and Liu et al.’s model [12], which achieve improved accuracy in estimating visual redundancies at the cost of higher complexity. A good review of JND models as well as approaches to exploit JND models in perceptual image coding was given by Wu et al. [13].

In this work, a new region-adaptive pixel-domain JND model based on efficient local operations is proposed for a more accurate detection of visibility thresholds compared with the classic JND model [9] and for a reduced complexity compared with more recent ones [11,12]. A low complexity pixel-domain perceptual image coder [14] is then used to exploit the visibility thresholds given by the proposed JND model. The coding algorithm addresses both coding efficiency and visual quality issues in conventional pixel-domain coders in a framework of adaptive downsampling guided by perceptual regions-of-interest (ROIs) based on JND thresholds. In addition, hardware architecture for both the proposed JND model and the perceptual encoder is presented. Experimental results including hardware resource utilization of FPGA-based implementations show reasonable performance and moderate hardware complexity for both the proposed JND model and the perceptual encoder. The remainder of the paper is organized as follows. Section 2 reviews background and existing work on pixel-domain JND modeling. The proposed JND model and its FPGA hardware architecture are presented in Section 3 and Section 4, respectively. Section 5 discusses the hardware architecture for the JND-based perceptual image coding algorithm [14]. Experimental results based on standard test images as well as FPGA synthesis results are presented in Section 6, which show the effectiveness of both the proposed JND model and the perceptual encoder. Section 7 summarizes this work.

## 2. Background in Pixel-Domain JND Modeling

**Figure 1:**
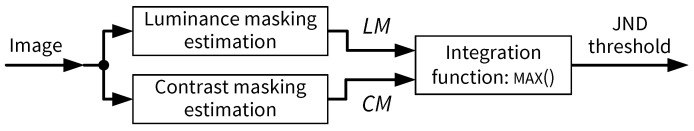
Chou and Li’s pixel-domain just-noticeable difference (JND) model (1995).

In 1995, Chou and Li proposed a pixel-domain JND model [9] based on experimental results of psychophysical studies. Figure 1 illustrates Chou and Li’s model. For each pixel location, two visual masking effects are considered, namely luminance masking and contrast masking, and visibility thresholds due to such effects are estimated based on functions of local pixel intensity levels. The two resulting quantities, luminance masking threshold LM and contrast masking threshold CM, are then combined by an integration function into the final JND threshold. In Chou and Li’s model, the integration takes the form of the MAX(·) function, i.e., the JND threshold is modeled as the dominating effect between luminance masking and contrast masking. Basic algorithmic parts of JND modeling described in the rest of this section are mainly based on Chou and Li’s model.

### 2.1. Luminance Masking Estimation

**Figure 2:**
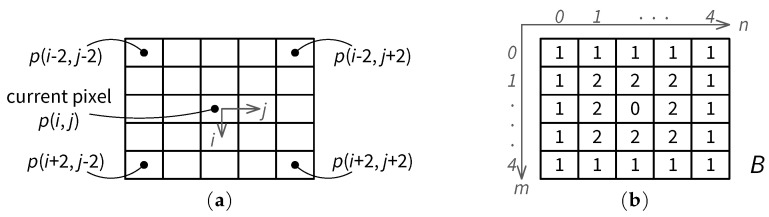
Pixel window for JND estimation and weighing factors for the background luminance: (**a**) JND estimation window of 5 × 5; and (**b**) weighing factor matrix *B*.

The luminance masking effect is modeled in [9] based on the average grey level within a 5 × 5 window centered at the current pixel location, as depicted in Figure 2a. Let BL(i,j) denote the background luminance at pixel location (i,j), with 0≤i<H and 0≤j<W for an image of size W×H. Let B(m,n) be a 5 × 5 matrix of weighing factors (m,n=0,1,2,3,4). As shown in Figure 2b, a relatively larger weight (2) is given to the eight inner pixels surrounding the current pixel, since such pixels have stronger influences on the average luminance at the current pixel location. The sum of all weighting factors in matrix *B* is 32. While other weighting factors can be considered for evaluating the average background luminance, the matrix *B* used in Chou and Li’s JND model [9] results in highly efficient computation and has been used in many subsequent models (see, e.g., [11,12]). Further, let p(i,j) denote the pixel grey level at (i,j). The average background luminance BL is then given by

(1) $$BL\left(i,j\right)=\frac{1}{32}\sum_{m=0}^{4}\sum_{n=0}^{4}p\;\;\left(i-2+m,\;\;j-2+n\right)\cdot B\left(m,n\right)$$

Obviously, Equation (1) can be implemented in hardware by additions and shifts only. It can be readily verified that 23 additions are required. Chou and Li examined the relationship between the background luminance and distortion visibility due to luminance masking based on results of subjective experiments [9,15], and concluded that the distortion visibility threshold decreases in a nonlinear manner as the background luminance changes from completely dark to middle grey (around 127 on an intensity scale from 0 to 255) and increases approximately linearly as the background luminance changes from grey to completely bright. Specifically, a square root function is used in [9] to approximate the visibility thresholds due to luminance masking for low background luminance (below 127), whereas and a linear function was used for high background luminance (above 127):

(2) $$LM\left(i,j\right)=\left\{\begin{array}{ll} T_{0}\cdot \left(1-\sqrt{\frac{BL(i,\;\;j)}{127}}\right)+3, & \mathrm{if}\;BL(i,j)\leq 127\\ \gamma \cdot \left(BL(i,j)-127\right)+3, & \mathrm{otherwise}, \end{array}\right.$$

where $T_{0}$ denotes the visibility threshold when the background luminance is 0 in the nonlinear region when BL(i,j)≤127, while γ is the slope of the growth of the visibility threshold in the linear region when the background luminance is greater than 127. The values of parameters $T_{0}$ and γ depend on the specific application scenario, such as viewing conditions and properties of the display. Both $T_{0}$ and γ increase as the viewing distance increases, leading to higher visibility thresholds. Default values of $T_{0}=17$ and $\gamma =\frac{3}{128}$ are used in [9], and these are also used for the JND model in this paper.

### 2.2. Contrast Masking Estimation

**Figure 3:**
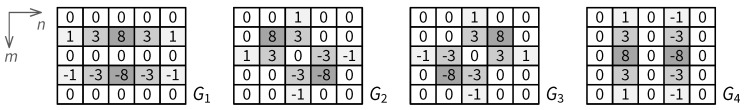
Directional intensity difference measurement operators $G_{1}$–$G_{4}$.

The contrast masking effect is modeled in [9] based on: (1) the background luminance at the current pixel; and (2) luminance variations across the current pixel in the 5×5 JND estimation window. Luminance variations, e.g., due to edges are measured by four spatial operators, $G_{1}$–$G_{4}$, as depicted in Figure 3. The result from an operator $G_{k}$ is the weighted luminance intensity difference across the current pixel in the direction corresponding to *k*, with k=1,2,3,4 for vertical, diagonal 135°, diagonal 45° and horizontal difference, respectively. The *k*th weighted luminance intensity difference $ID_{k}$ is calculated by 2D correlation, and the maximum weighted luminance difference MG is obtained as:

(3) $$\begin{array}{cc} ID_{k}\left(i,j\right) & =\frac{1}{16}\sum_{m=0}^{4}\sum_{n=0}^{4}p\;\;\left(i-2+m,\;\;j-2+n\right)\cdot G_{k}\left(m,n\right) \end{array}$$

(4) $$\begin{array}{cc} MG(i,j) & =\underset{k=1,2,3,4}{\mathrm{MAX}}\left\{\left|ID_{k}\left(i,j\right)\right|\right\} \end{array}$$

In Chou and Li’s model, for a fixed average background luminance, the visibility threshold due to contrast masking is a linear function of MG (also called luminance edge height in [9]) by

(5) CM(i,j)=α(i,j)·MG(i,j)+β(i,j)

Both the slope α and intercept β of such a linear function depend on the background luminance BL. The relationship between α,β and BL was modeled by Chou and Li as

(6) $$\begin{array}{cc} \alpha (i,j) & =BL(i,j)\cdot 0.0001+0.115 \end{array}$$

(7) $$\begin{array}{cc} \beta (i,j) & =\lambda -BL(i,j)\cdot 0.01\;\; \end{array}$$

Parameter λ in Equation (7) depends on the viewing condition. The value of λ increases as the viewing distance becomes larger, leading to higher visibility thresholds. A default value of λ=0.5 is used in [9].

### 2.3. Formulation of JND Threshold

In Chou and Li’s model, the final JND threshold is considered to be the dominating effect between luminance masking and contrast masking:

(8) $$JND\left(i,j\right)=\mathrm{MAX}\;\;\left\{LM(i,j),CM(i,j)\;\;\right\}$$

Since in real-world visual signals there often exist multiple masking effects simultaneously, such as luminance masking and contrast masking, the integration of multiple masking effects into a final visibility threshold for the HVS is a fundamental part of a JND model [11]. Contrary to Chou and Li, who considered only the dominating effect among different masking effects, Yang et al. [11,16] proposed that: (1) in terms of the visibility threshold, the combined effect *T* in the presence of multiple masking effects $T^{1},T^{2},\ldots ,T^{N}$ is greater than that of a single masking source $T^{i}\;\;$ (i=1,2,…,N); and (2) the combined effect *T* can be modeled by a certain form of addition of individual masking effects, whereas *T* is smaller than a simple linear summation of the individual effects $T^{i},i=1,2,\ldots ,N$, i.e.,

(9) $$\mathrm{MAX}\left\{T^{1},T^{2},\ldots ,T^{N}\right\}<T<\sum_{i=1}^{N}T^{i}\;\;$$

Yang et al. [11] further proposed that the right-hand side of the above inequality is due to the overlapping of individual effects. A pair-wise overlap $O^{i,\;\;j}$ is hence modeled for the combination of two individual masking factors $T^{i},T^{j}\;\left(i<j\right)$ by a nonlinear function $\gamma (T^{i},T^{j})$, weighted by an empirically determined gain reduction coefficient $C^{i,\;\;j}$ (0<C<1), i.e.,

(10) $$O^{i,\;\;j}=C^{i,\;\;j}\cdot \gamma \left(T^{i},T^{j}\right)\;\;$$

The total overlap is modeled as the sum of overlaps between any pair of masking factors. The combined visibility threshold is given by the difference between the sum of all thresholds due to individual masking effects and the total overlap, called the nonlinear-additivity model for masking (NAMM) [11]:

(11) $$T=\sum_{i=1}^{N}T^{i}-\sum_{i=1}^{N}\sum_{j=i+1}^{N}O^{i,\;\;j}=\sum_{i=1}^{N}T^{i}-\sum_{i=1}^{N}\sum_{j=i+1}^{N}C^{i,\;\;j}\cdot \gamma \left(T^{i},T^{j}\right)$$

For simplicity and the compatibility with existing models including Chou and Li’s, in Yang et al.’s model [11] the nonlinear function γ is approximated as the minimum function MIN(·), and only luminance masking and contrast masking effects are considered. The result is therefore an approximation of the general model given by Equation (11). In Yang et al.’s model, the final visibility threshold at pixel location (i,j) in component θ (θ=Y,Cb,Cr) of the input image is a nonlinear combination of the luminance masking threshold $T^{L}$ and an edge-weighted contrast masking threshold $T_{\theta }^{C}$ given by

(12) $$JND_{\theta }\left(i,j\right)=T^{L}\left(i,j\right)+T_{\theta }^{C}\left(i,j\right)-C_{\theta }^{L,C}\cdot \mathrm{MIN}\left\{T^{L}\left(i,j\right),T_{\theta }^{C}\left(i,j\right)\right\}\;\;$$

Yang et al. selected default values of gain reduction coefficients as $C_{Y}^{L,C}=0.3$, $C_{Cb}^{L,C}=0.25$ and $C_{Cr}^{L,C}=0.2$ based on subjective tests in [16]. The compatibility with Chou and Li’s model can be seen by letting θ=Y and $C_{Y}^{L,C}=1$ in Equation (12), i.e., considering the luminance image only and assuming maximum overlapping between the luminance and contrast masking effects.

## 3. Proposed JND Model

**Figure 4:**
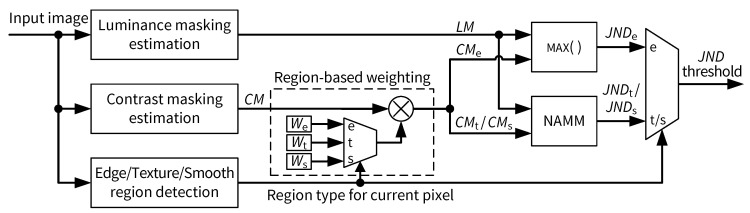
Block diagram of the proposed JND model.

In the proposed JND model, each input pixel is assumed to belong to one of three basic types of image regions: edge (e), texture (t) and smoothness (s). The weighting of the contrast masking effect, as well as the combination of the basic luminance masking threshold (LM) and contrast masking threshold (CM) into the final JND threshold, is dependent on the region type of the current pixel. Figure 4 illustrates the proposed JND model, where $W_{e}$, $W_{t}$ and $W_{s}$ are factors used for weighting the contrast masking effect in edge, texture and smooth regions, respectively. As shown in Figure 4, to combine LM and weighted CM values, the MAX() function is used for edge and NAMM is used for texture and smooth regions. Depending on the region type of a current pixel, the final output, i.e., JND threshold for the current pixel, is selected from three candidates $JND_{e}$, $JND_{t}$ and $JND_{s}$, corresponding to the visibility threshold evaluated for the edge, texture and smooth region, respectively.

The individual treatment of edge regions in a JND model was first proposed by Yang et al. [16]. Clear edges such as object boundaries are familiar to the human brain, since they typically have simple structures and draw immediate attention from an observer. Hence, even a non-expert observer can be considered as relatively “experienced” in viewing edge regions of an image. As a result, distortions, e.g., due to lossy compression, are more easily identified at edges than in other regions with luminance non-uniformity [11,17,18]. In Yang et al.’s work [11], visibility thresholds due to contrast masking are reduced for edge regions (detected by the Canny operator) compared with non-edge regions. Weighting factors of 0.1 and 1.0 are used for edge and non-edge pixels, respectively, such that edges are preserved in a subsequent compression encoder exploiting the JND thresholds.

Textures, on the other hand, are intensity level variations usually occurring on surfaces, e.g., due to non-smoothness of objects such as wood and bricks. Since textures have a rich variety and generally exhibit a mixture of both regularity (e.g., repeated patterns) and randomness (e.g., noise-like scatterings) [19], the structure of a texture is much more difficult to predict than that of an edge for the human brain. Eckert and Bradley [18] indicated that about three times the quantization noise can be hidden in a texture image compared with an image of simple edges with similar spectral contents. To adequately estimate the contrast masking effects in texture regions, Liu et al. [12] proposed to decompose the image into a textural component and a structural one. Both components are processed independently for contrast masking in Liu et al’s model [12], with the masking effects computed for the textural and structural components weighted by factors of 3 and 1, respectively. The masking effects of both components are added up to obtain the final contrast making in Liu et al.’s JND model.

The main differences of our JND model to the works by Chou and Li [9], Yang et al. [11] and Liu et al. [12] are: (1) marking pixels in an input image as edge, texture or smooth regions, instead of decomposing the image into multiple components processed separately; (2) combination of LM and CM into the final JND threshold using the maximum operator for edge regions and NAMM [11] for non-edge regions; (3) alternative weighting of the contrast masking effect compared with [11,12]; and (4) less complex edge and texture detection schemes more suitable for FPGA implementation compared with [11,12]. The following subsections provide details on our JND model.

### 3.1. Edge and Texture Detection

**Figure 5:**
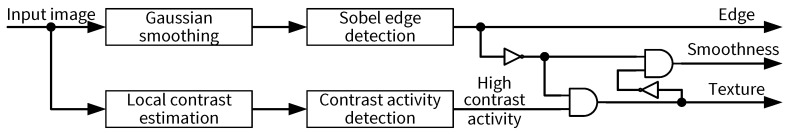
Edge, texture and smooth region detection scheme.

**Figure 6:**
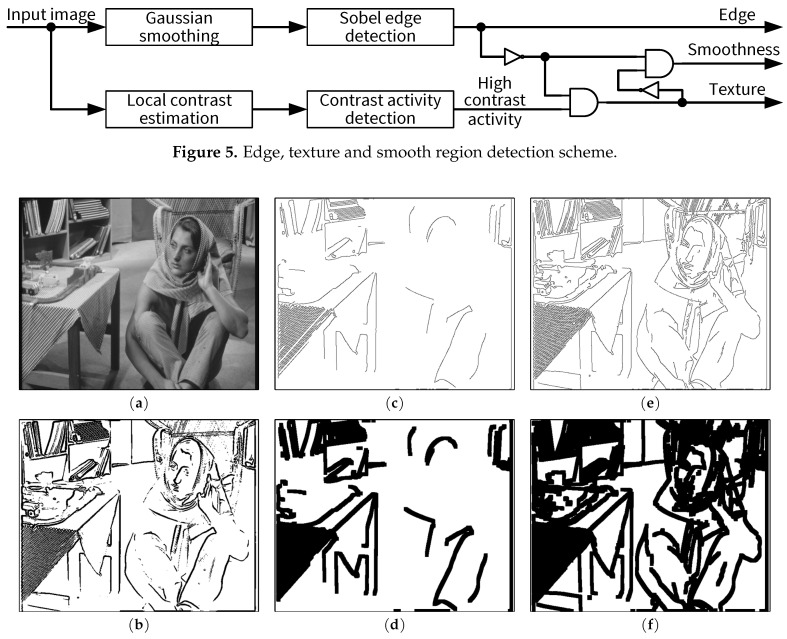
Edges (black) obtained in the proposed and Yang et al.’s JND model: (**a**) original BARB image; (**b**) edges detected by the proposed scheme with default edge-magnitude threshold 11; (**c**) output of original Canny with edge sensitivity threshold 0.5 (default in [11]); (**d**) actual edge regions from Yang et al.’s implementation [22] with threshold 0.5; (**e**) original Canny with edge sensitivity threshold 0.25; and (**f**) actual edge regions from Yang et al.’s implementation [22] with edge sensitivity threshold 0.25.

Each input pixel is assigned one out of three possible regions in the input image, i.e., edge, texture and smoothness. Different regions are detected by lightweight local operations such as 2D filtering, which can be implemented efficiently on FPGAs (see Section 4). Figure 5 illustrates the detection scheme, where the input is the original image while the outputs are three binary maps corresponding to edge, texture and smooth regions, respectively. Edges are detected by the Sobel operator [20] which uses two 3×3 kernels. It is well known that the Sobel operator requires less computation and memory compared with the Canny operator [21], which is used in the JND models in [11,12]. To reduce the impact of noise in the input image, Gaussian low-pass filtering is performed prior to edge detection. A two-dimensional 3×5 Gaussian kernel with standard deviation σ=0.83 is used by default in the proposed JND model. The vertical size of the Gaussian kernel is chosen as 3 for a low memory requirement as well as a low latency of an FPGA implementation. For computational efficiency, an integer approximation of the Gaussian kernel discussed in Section 6.1 is used, which can be implemented efficiently by shifts and additions. Figure 6 presents edges detected in different JND models for the BARB test image. Edges obtained by the proposed lightweight scheme (i.e., Gaussian smoothing followed by Sobel) are depicted in Figure 6b. The four panels in the middle and right columns of Figure 6 show outputs of the Canny edge detector in Yang et al.’s model [11] with sensitivity thresholds of 0.5 (default [11], middle panels) and 0.25 (right panels). Morphological operations have been used in Yang et al.’s software implementation [22] of their JND model to expand the edges given by the original Canny operator (see Figure 6d,f). Such operations result in bigger regions around the edges having reduced visibility thresholds to protect edge structures.

Many of the well-known texture analysis techniques (e.g., [23]) focus on distinguishing between different types of textures. While such techniques achieve promising results for image segmentation, they typically require larger blocks and computationally-intensive statistical analysis such as multi-dimensional histograms, and their complexity/performance trade-offs are not well-suited for JND modeling especially in resource-constrained scenarios. As discussed earlier, a desirable property of a JND model is to distinguish textures as opposed to structural edges and smooth regions, and a reasonable complexity/quality trade-off is an advantage especially for FPGA applications. Even if some texture regions were not picked up by a lightweight texture detection scheme compared with a sophisticated one, the visibility thresholds in such regions computed by the JND model would still be valid, e.g., for a visually lossless compression of the input image, since weighting factors for contrast masking are generally smaller in non-texture regions than in texture ones. For the reasons above, a low complexity local operator is used for texture detection in our JND model.

**Figure 7:**
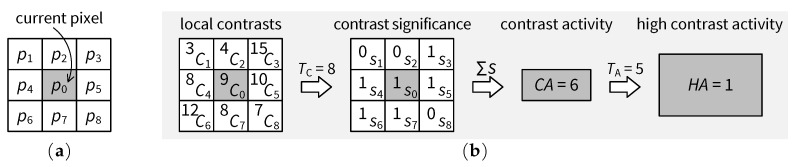
Illustration of contrast activity detection: (**a**) neighborhood for local contrast estimation; and (**b**) example of local contrasts, contrast significance and derivation of the high-contrast-activity decision.

The proposed texture detection scheme works as follows. Firstly, a local contrast value is calculated for every pixel location. Figure 7a shows a 3×3 neighborhood for evaluating the local contrast, where $p_{0}$ is the intensity value at the current pixel location and $p_{1}$–$p_{8}$ are intensity values of the eight immediate neighbors of $p_{0}$. Let μ be the average of all intensity values in the 3 × 3 neighborhood. Then, the local contrast *C* can be measured for the current pixel location in terms of mean absolute deviation (MAD):

(13) $$C^{\mathrm{MAD}}=\frac{1}{9}\sum_{i=0}^{8}\left|p_{i}-\mu \right|,\;\mathrm{where}\;\mu =\frac{1}{9}\sum_{j=0}^{8}p_{j}\;\;$$

Obviously, $C^{\mathrm{MAD}}$ is invariant to image rotation and intensity-level shifts. In an implementation, e.g., based on FPGA, the divisions in Equation (13) can be avoided since such divisions can be canceled by multiplications on both sides of the equation. A division-free implementation of the local contrast calculation equivalent to that in Equation (13) is used in the proposed hardware architecture for the JND model, as discussed in Section 4.4.2.

Next, the total contrast activity in the neighborhood is estimated based on local contrasts. Figure 7b presents an example of computed local contrasts, the thresholding of such local contrasts into a contrast significance map, the computation of a contrast activity value and finally the derivation of a binary high-contrast-activity decision. Let $C_{i}$ be the local contrast at pixel location *i* in the 3×3 neighborhood centered about the current pixel. Then, contrast significance $s_{i}$ is given by

(14) $$s_{i}=\left\{\begin{array}{ll} 1, & \mathrm{if}\;C_{i}\geq T_{C}\\ 0, & \mathrm{otherwise}, \end{array}\right.$$

where $T_{C}$ is a threshold for local contrast. A higher value of $T_{C}$ corresponds to a smaller number of local contrasts detected as significant. In this paper, $T_{C}=8$ is used. Contrast activity CA at the current pixel location is estimated as the total number of significant local contrasts in the 3×3 neighborhood:

(15) $$CA=\sum_{i=0}^{8}s_{i}\;\;$$

**Figure 8:**
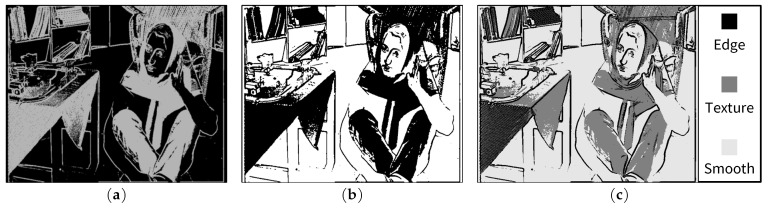
Texture information of the BARB image in the proposed scheme; (**a**) visualization of contrast activity (treated as grey values and multiplied by 20 for visibility); (**b**) high contrast activity (black) regions after thresholding with $T_{A}=5$; and (**c**) final edge, texture and smooth regions.

The presence of a texture is typically characterized by a high contrast activity (HA):

(16) $$HA=\left\{\begin{array}{cc} 1, & \mathrm{if}\;CA\geq T_{A}\\ 0, & \mathrm{otherwise}, \end{array}\right.$$

where $T_{A}$ is a threshold for contrast activity. A lower value of $T_{A}$ corresponds to a higher sensitivity to local contrast activities. In this paper, $T_{A}=5$ is used. Figure 8a plots the contrast activities computed for the BARB image (cf. Figure 6a). The HA map after thresholding is shown in Figure 8b.

Finally, denoting the binary output of the edge detector by *E*, a pixel is considered to be in a texture region (*T*) if it has a high contrast activity and is not an edge, as indicated in Figure 5:

(17) $$T=HA\wedge \bar{E}$$

and a pixel is considered to be in a smooth region (*S*) if it is neither an edge nor a texture:

(18) $$S=\bar{E}\wedge \bar{T}$$

The final edge, texture and smooth regions obtained for the BARB image are depicted in Figure 8c. While it is possible to achieve a better separation of the image into different regions using more sophisticated texture analysis and segmentation algorithms such as in Liu et al.’s model [12], the proposed lightweight edge and texture detection scheme has achieved quite reasonable results, as shown in Figure 8c, which provides a firm basis for a region-based weighting of contrast masking discussed in the next subsection. Comparisons of different JND models are given in Section 6.2 and Section 6.3.

### 3.2. Region-Based Weighting of Visibility Thresholds due to Contrast Masking

In the proposed JND model, each basic contrast masking threshold estimated using Equation (5) is multiplied by a weighting factor based on the region in which the current pixel is located. Let $W_{e}$, $W_{t}$ and $W_{s}$ be the weighting factors for edge (e), texture (t) and smooth (s) regions, respectively. Then, the adaptively weighted contrast masking effect $CM_{\kappa }$ is given by

(19) $$CM_{\kappa }\left(i,j\right)=W_{\kappa }\cdot CM\left(i,j\right),\;\kappa =\left\{e,t,s\right\}$$

where κ denotes the region type of the current pixel. In Yang et al.’s JND model [11], a weighting factor equivalent to $W_{e}=0.1$ is used to preserve visual quality in edge regions, while in Liu et al.’s JND model [12] a weighting factor equivalent to $W_{t}=3$ is used to avoid underestimating visibility thresholds in texture regions. From Equation (19), it is obvious that larger values of $W_{e}$, $W_{t}$ and $W_{s}$ correspond to larger results for the contrast masking effects (and hence the final JND thresholds) in edge, texture and smooth regions, respectively. Values for weighting factors $W_{e}$, $W_{t}$ and $W_{s}$ may vary, for example depending on different viewing conditions and applications. Based on our experiments as well as for reasons discussed in the following subsection, values for the weighting factors are selected as $W_{e}=1$, $W_{t}=1.75$ and $W_{s}=1$ in this work as default for the proposed JND model for normal viewing conditions and general purpose test images. More details about the test images and viewing conditions in our experiments are provided in Section 6.2.

### 3.3. Final JND Threshold

In the proposed JND model, the luminance masking and weighted contrast masking effects are combined using the NAMM model in texture (t) and smooth (s) regions, whereas, in edge (e) regions, the masking effects are combined using the maximum operator MAX(·), as shown in Equation (20).

(20) $$JND\left(i,j\right)=\left\{\begin{array}{ll} LM\left(i,j\right)+CM_{\kappa }\left(i,j\right)-C_{Y}^{L,C}\cdot \mathrm{MIN}\left\{LM\left(i,j\right),CM_{\kappa }\left(i,j\right)\right\}, & \mathrm{if}\;\kappa =\{t,s\}\\ \mathrm{MAX}\{LM\left(i,j\right),CM_{e}\left(i,j\right)\}, & \mathrm{otherwise}. \end{array}\right.$$

The individual treatment of edge regions is based on the similarity between simple edge regions and scenarios in classical psychophysical experiments to determine distortion visibility thresholds in the presence of luminance edges, where simple edges are studied under different background luminance conditions [8]. Hence, for well-defined edges, the visibility thresholds modeled by Chou and Li based on such experiments should be considered as suitable. For the same reason, we selected $W_{e}=1$.

## 4. Hardware Architecture for the Proposed JND Model

### 4.1. Overview of Proposed JND Hardware Architecture

**Figure 9:**
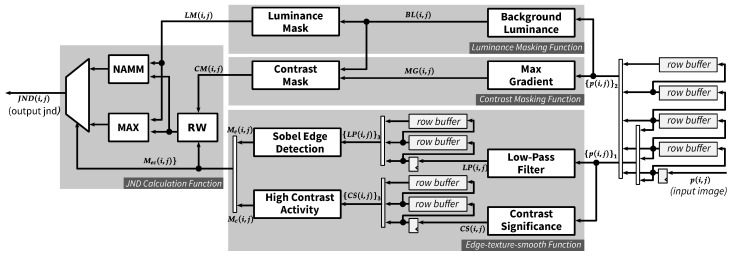
Overall architecture of the proposed JND model.

Figure 9 depicts the overall hardware architecture of proposed JND estimation core implemented on FPGA. The core includes four main parts (names of functional modules of the architecture are indicated in italics): *Luminance Masking Function*, *Contrast Masking Function*, *Edge-texture-smooth Function*, and *JND Calculation Function*. The streaming input pixel (p(i,j)) is first buffered in row buffers which are needed for the filtering operations applied in our JND model. From the row buffers, pixels are grouped as a column of 3 pixels (${\left\{p(i,j)\right\}}_{1}$) or a column of 5 pixels (${\left\{p(i,j)\right\}}_{2}$). The 3-pixel column is sent to the *Edge-texture-smooth Function*, while the 5-pixel column is sent to both *Luminance Masking Function* and *Contrast Masking Function*. From these three functions, region mask $M_{\mathrm{ec}}\left(i,j\right)$, luminance masking threshold LM(i,j) and contrast masking threshold CM(i,j) are calculated, respectively. The *JND Calculation Function* combines these masks together and generates the final JND value (JND(i,j)) for each pixel in the input image.

#### 4.1.1. Row Buffer

The proposed JND architecture employs a common row buffer design [24], which includes registers for the current row pixel and several FIFOs for previous row pixels. Suppose *r* is the vertical window radius of a filter kernel, the number of required FIFOs for this design is 2·r−1. The row buffers are needed before every filtering operation. In our implementation, there are three places where row buffers are deployed: after the input, before the calculation of high contrast activity and after low-pass filtering. The latter two row buffers are for r=1 and the first row buffer is for r=1 and r=2.

As shown in Figure 9, the rightmost row buffers contain four FIFOs to support a filter kernel with a maximum size of 5 (r=2). The output of the row buffer forms a pixel-array denoted as ${\left\{p(i,j)\right\}}_{2}$ (see Equation (21)) which is fed to *Background Luminance* module and *Max Gradient* module where 5×5 filter kernels are applied. A subset of this row buffer output, ${\left\{p(i,j)\right\}}_{1}$, is sent to *Low-Pass Filter* module and *Contrast Significance* module which consist of 3×5 and 3×3 kernel filtering operations, respectively.

(21) $${\left\{p(i,j)\right\}}_{r}=\left\{p\left(i-r,j\right),p\left(i-r+1,j\right),\ldots ,p\left(i+r-1,j\right),p\left(i+r,j\right)\right\}$$

#### 4.1.2. Pipelined Weighted-Sum Module

**Figure 10:**
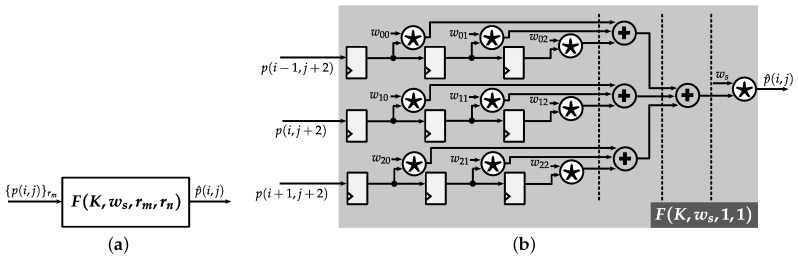
Pipelined Weighted-Sum (PWS) module. (**a**) Block representation. (**b**) PWS for 3 × 3 kernel. Dotted lines indicate possible pipeline cuts. The ★ operator indicates customized shift-based multiplier.

For filtering operations, which are employed in several parts of proposed JND model, a common design to perform weighted-sum is introduced, as illustrated in Figure 10. The block representation of a *Pipelined Weighted-Sum* (*PWS*) module is depicted in Figure 10a. The input to this module is an array of column pixel denoted as ${\left\{p(i,j)\right\}}_{r_{m}}$, and the output is a weighted-sum value calculated as

(22) $$\hat{p}\left(i,j\right)=w_{s}\cdot \left(\sum_{m=0}^{2\cdot r_{m}-1}\;\sum_{n=0}^{2\cdot r_{n}-1}\left(w_{mn}\cdot p\left(i+m-r_{m},j+n-r_{n}\right)\right)\right).$$

The *PWS* module is parameterized as a function $F(K,w_{s},r_{m},r_{n})$, where *K* is a 2D array of coefficients, $w_{s}$ is an output scaling factor, and $r_{m},r_{n}$ are vertical and horizontal kernel window radius, respectively. Figure 10b presents a zoom-in sample design for $F(K,w_{s},1,1)$ with *K* defined as

(23) $$K=\left[\begin{array}{ccc} w_{00} & w_{01} & w_{02}\\ w_{10} & w_{11} & w_{12}\\ w_{20} & w_{21} & w_{22} \end{array}\right]$$

The operator denoted as ★ is a *Customized Shift-based Multiplier* (*CSM*), which generally consists of sum and shift operators. The actual content of this operator will be defined according to the value of a given coefficient. For example, considering the coefficient −3 in kernel $G_{1}$ (see Figure 3), the multiplication of this coefficient with a pixel value *p* can be rewritten as: −3·p=−(p<<1+p), which now consists of one left shift operator, one adder and one sign-change operator. Since all the coefficients are known, this customized multiplier strategy allows us to optimize for both timing and hardware resource.

### 4.2. Luminance Masking Function

**Figure 11:**
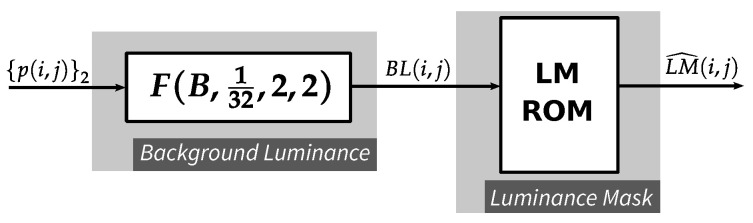
Luminance masking function.

As discussed in Section 2.1, the calculation of the luminance masking threshold (LM) includes two steps. The first step is finding the background luminance (BL), which can be realized by a *PWS* module $F(B,\frac{1}{32},2,2)$. The second step is calculating LM based on the value of BL. Since the value of BL belongs to the same range as of input pixel value, which is an 8-bit integer in our implementation, the latter step can be simply realized as a look-up operation (see Figure 11). The *LM ROM* is implemented by Block RAM and has 256 entries, each with 5+σ bits where 5 and σ are implicitly the number of bits for integer part and fractional part of LM, respectively. The output of this function is indeed $2^{\sigma }$ larger than the actual value of LM ($\hat{LM}\left(i,j\right)=2^{\sigma }\cdot LM\left(i,j\right)$). The scaling factor $2^{\sigma }$ is discussed further in Section 4.3.

### 4.3. Contrast Masking Function

**Figure 12:**
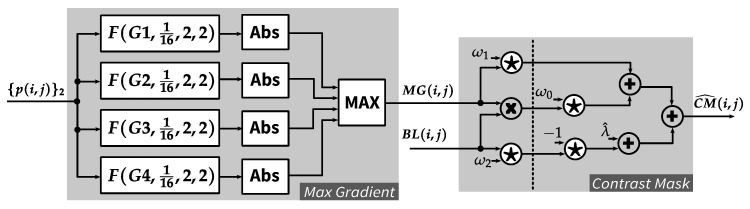
Contrast masking function.

Contrast masking function consists of two modules: the first module (*Max Gradient*) calculates MG based on input pixels from the row buffer. The second module (*Contrast Mask*) computes CM from MG and BL, which is the output of *Background Luminance* module (see Figure 12). For each of the directional gradient operations ($G_{i},\;i=1,2,3,4$), *PWS* module is deployed with output scaling factor $w_{s}=\frac{1}{16}$ and the two radii are set to 2. Absolute values of these modules’ outputs are then calculated, by *Abs* functions, and compared to each other to find the maximum value (MG). The absolute function can be simply realized by a multiplexer with *select* signal being the most significant bit of the input.

The contrast masking threshold (CM) is calculated for each pixel location based on the value of MG and BL. This calculation requires multiplications by several real numbers which cannot be accurately converted to shift-based operators. To keep the implementation resource-efficient, without using floating point operations, a fixed-point based approximation strategy is proposed as in Equation (24). A scaling factor $2^{\sigma }$ is applied to the overall approximation of the given real numbers for providing more accuracy adjustment.

(24) $$\begin{array}{ll} \omega_{0}=2^{\sigma }\cdot \left(2^{-14}+2^{-15}+2^{-17}\right)\approx 2^{\sigma }\cdot \;0.0001 & \omega_{2}=2^{\sigma }\cdot \left(2^{-7}+2^{-9}\right)\approx 2^{\sigma }\cdot \;0.01\\ \omega_{1}=2^{\sigma }\cdot \left(2^{-3}-2^{-7}-2^{-9}\right)\approx 2^{\sigma }\cdot \;0.115 & \hat{\lambda }=2^{\sigma }\cdot 2^{-1} \end{array}$$

With the above approximations, Equations (5)–(7) are then rewritten as Equation (25) and implemented as *Contrast Mask* module shown in Figure 12. In this implementation, σ is empirically set to 5, since it provides a reasonable trade-off between accuracy and resource consumption.

(25) $$\hat{CM}\left(i,j\right)=BL\left(i,j\right)\cdot MG\left(i,j\right)\cdot \omega_{0}+MG\left(i,j\right)\cdot \omega_{1}+\hat{\lambda }-BL\left(i,j\right)\cdot \omega_{2}$$

### 4.4. Edge-Texture-Smooth Function

This function consists of two separate modules: *Edge Detection* and *High Contrast Activity* which, respectively, mark pixel location belonging to edge region and high contrast activity region. These modules receive the same 3-pixel column as an input and output a binary value for each pixel location. The output of *Edge Detection* module ($M_{e}\left(i,j\right)$) and *High Contrast Activity* module ($M_{c}\left(i,j\right)$) are combined into a two-bit signal ($M_{\mathrm{ec}}\left(i,j\right)$), which has $M_{e}\left(i,j\right)$ as the most significant bit (MSb) and $M_{c}\left(i,j\right)$ as the least significant bit (LSb). $M_{\mathrm{ec}}\left(i,j\right)$ is then used as the *select* signal for multiplexers in *JND Calculation Function*. The following subsections discuss each of these modules in detail.

#### 4.4.1. Edge Detection

**Figure 13:**
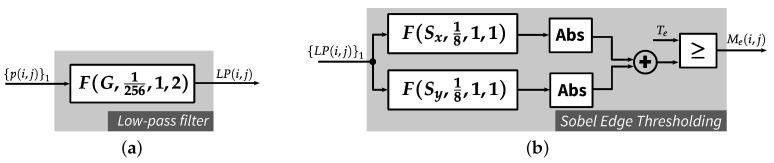
Edge detection module: (**a**) low-pass filter; and (**b**) Sobel edge thresholding module.

The edge detection algorithm applied in the proposed JND model requires three filtering operations: one for Gaussian filtering and the other two for finding the Sobel gradients in horizontal and vertical directions. These filters are realized by *PWS* modules, as depicted in Figure 13a,b. The coefficient array *G* can be found in Section 6.1, and the kernels $S_{x},S_{y}$ are as follows:

(26) $$\begin{array}{l} S_{x}=\left[\begin{array}{ccc} -1 & 0 & 1\\ -2 & 0 & 2\\ -1 & 0 & 1 \end{array}\right] \end{array}\;\begin{array}{l} S_{y}=\left[\begin{array}{ccc} -1 & -2 & -1\\ 0 & 0 & 0\\ 1 & 2 & 1 \end{array}\right] \end{array}$$

#### 4.4.2. High Contrast Activity

**Figure 14:**
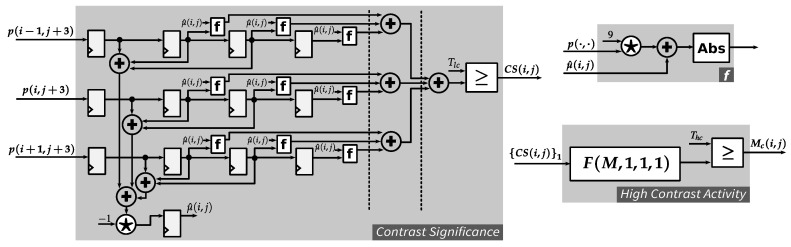
High-contrast activity module: (**Left**) contrast significance estimation module; (**Top-right**) function *f*; and (**Bottom-right**) high contrast activity thresholding module.

To detect high contrast activity regions, the contrast significance CS needs to be calculated for each pixel location. The proposed architecture for this task is illustrated in Figure 14. Considering Equation (13), two divisions by 9 are required for finding $C^{\mathrm{MAD}}$. This can actually introduce some errors to the implementation using fixed-point dividers. Therefore, the following modification is done to find CS:

(27) $${\hat{C}}^{\mathrm{MAD}}=\sum_{j=0}^{8}\left|9\cdot p_{j}+\hat{\mu }\right|,\;\mathrm{where}\;\hat{\mu }=-\sum_{j=0}^{8}p_{j}\;\;$$

It is obvious that the value of ${\hat{C}}^{\mathrm{MAD}}$ is 81 times as large as $C^{\mathrm{MAD}}$. Therefore, instead of comparing $C^{\mathrm{MAD}}$ to the threshold $T_{C}$ as in Equation (14), the modified ${\hat{C}}^{\mathrm{MAD}}$ is now compared to the new threshold $T_{\mathrm{hc}}=81\cdot T_{C}$. This strategy indeed requires extra hardware resources if $T_{C}$ is not implemented as a constant but can guarantee the accuracy of CS without using floating-point operation.

Considering the implementation of *Contrast Significance* module depicted in Figure 14, the input 3-pixel column is registered four times: the first three register columns are for calculating $\hat{\mu }$ and the last three register columns are for calculating ${\hat{C}}^{\mathrm{MAD}}$. There is one clock cycle delay between these two calculations, which is resolved by inserting a register, as shown in the bottom-left side of the module.

### 4.5. JND Calculation Function

**Figure 15:**
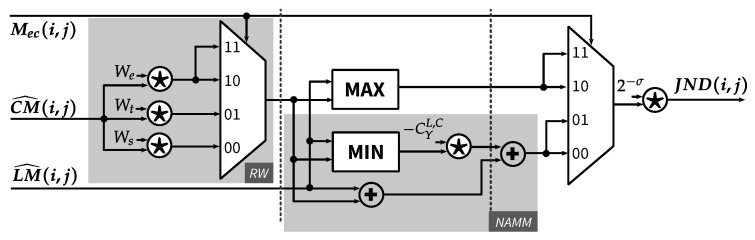
JND calculation function.

Figure 15 presents the implementation of Equations (19) and (20), which calculate the final value of JND based on the contrast masking threshold ($\hat{CM}$), the luminance masking threshold ($\hat{LM}$) and the region mask ($M_{\mathrm{ec}}$). The *Region-based Weighting* module (*RW*) applies a weighting factor to the incoming contrast mask. The weighting factors, which depend on the region type for the current pixel, are $W_{e}=1$, $W_{t}=1.75$ and $W_{s}=1$ for edge, texture and smooth regions, respectively. The texture weight can be rewritten as $W_{t}=2^{1}-2^{-2}$, which results in two shift operations and one adder in our customized shift-based multiplier. The other two weights can be simply realized as wires connecting the input and the output. The region mask is used as the *select* signal of a multiplexer in order to choose correct weighted value for the next calculation phase.

In the next calculation phase, the weighted contrast masking threshold (${\hat{CM}}_{\kappa }$) is fed to the *MAX* module and *NAMM* module, which compute the JND value for the edge region and non-edge regions, respectively. For the *CSM* module in *NAMM*, an approximation is done for $C_{Y}^{L,C}$, as shown in Equation (28). The final value of JND is then computed by removing the scaling factor $2^{\sigma }$ applied to the input contrast masking and luminance masking thresholds.

(28) $${\hat{C}}_{Y}^{L,C}=2^{-2}+2^{-5}+2^{-6}+2^{-8}\approx 0.3$$

## 5. JND-Based Pixel-Domain Perceptual Image Coding Hardware Architecture

**Figure 16:**
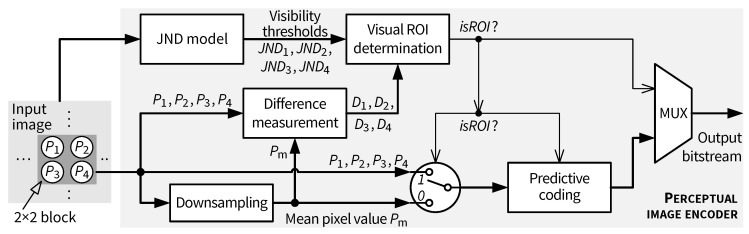
JND-based pixel-domain perceptual image coding algorithm proposed in [14].

A low complexity pixel-domain perceptual image coding algorithm based on JND modeling has been proposed in our earlier work [14]. Its principle is briefly described in what follows, before addressing architectural aspects. The perceptual coding algorithm is based on predictive coding of either the downsampled pixel value or the original pixels according to the encoder’s decision about whether the downsampled pixel is sufficient to represent the corresponding original pixels at visually lossless (or at least visually optimized in the case of suprathreshold coding) quality. Figure 16 illustrates the algorithm of the perceptual encoder. The *Visual ROI determination* block compares local distortions due to downsampling against the distortion visibility thresholds at corresponding pixel locations given by the pixel-domain JND model. If any downsampling distortion crosses the JND threshold, the current downsampling proximity (a 2 × 2 block in [14]) is considered to be a region-of-interest, and all pixels therein are encoded. In non-ROI blocks, only the downsampled mean value is encoded. In both cases, the encoder ensures that the difference from a decoded pixel to the original pixel does not exceed the corresponding JND threshold, fulfilling a necessary condition on visually lossless coding from the perspective of the JND model. The predictive coder exploits existing low complexity algorithmic tools from JPEG-LS [25] such as pixel prediction, context modeling and limited-length Golomb coding but uses a novel scan order so that coherent context modeling for ROI and non-ROI pixels is possible. The ROI information and the predictive coder’s outputs are combined to form the output bitstream. More detailed information on the coding algorithm can be found in [14]. The remainder of this section provides information on the hardware architecture for such a perceptual encoder.

### 5.1. Top-Level Architecture of the JND-Based Pixel-Domain Perceptual Encoder

**Figure 17:**
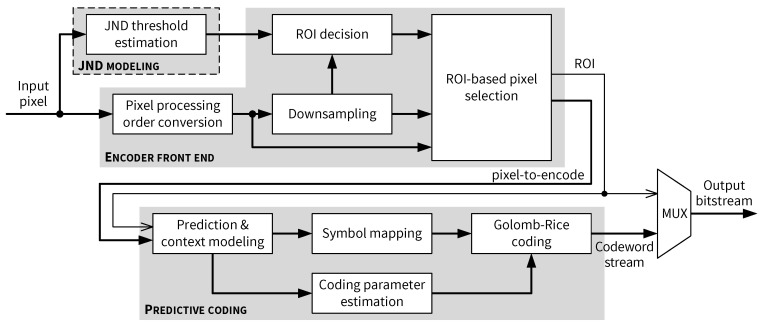
Overview of the proposed JND-based perceptual encoder architecture.

The overall proposed architecture for the perceptual encoder is depicted in Figure 17. On the top level, apart from the JND module discussed in Section 4, the proposed encoder architecture can be divided into two main parts: an *Encoder front end* module and a *Predictive coding* module. As shown in Figure 17, pixels encoded by the predictive coding path are provided by the *Encoder front end*, which performs the following tasks:

- Generate the skewed pixel processing order described in [14].
- Downsample the current 2×2 input block.
- Determine whether the current input 2×2 block is an ROI based on the JND thresholds.
- Select the pixel to be encoded by the predictive coding path based on the ROI status.

For clarity, the JND module, as well as the delay element for synchronizing the JND module outputs with the input pixel stream for the encoder, is omitted from the discussions on the encoder architecture in the rest of the paper. In addition, since existing works (e.g., [26]) have well covered architectural aspects of fundamental pixel-domain predictive coding algorithms such as JPEG-LS, the following discussion focuses mainly on the aspects of the proposed encoder architecture that enable the skewed pixel processing, the JND-based adaptive downsampling and the ROI-based pixel selection [14].

### 5.2. Input Scan Order vs. Pixel Processing Order

**Figure 18:**
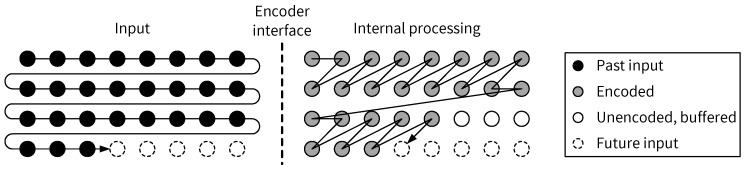
Input pixel scan order (raster scan) vs. internal pixel processing order (skewed scan [14]).

The raster scan order represents a common sequence in which pixels in an image are produced or visited, for example at the output interface of a sensor or at the input interface of an encoder. The encoder architecture in this paper assumes that pixels of an input image are streamed sequentially into the encoder in a raster scan order, with the source of the input image being arbitrary, such as a camera sensor, e.g., when the encoder is directly connected to the sensor to compress raw pixels, or an external memory, e.g., when the whole image needs to be temporarily buffered for denoising before compression. Inside the encoder, pixels do not have to be processed in the same order as they have been received. Figure 18 shows an example in which the input pixels are received in a raster scan order whereas the actual encoding of the pixels follows a skewed scan order [14]. Obviously, internal pixel buffers such as block RAMs on FPGAs are required, if an encoder’s internal pixel processing order differs from its input pixel scan order. An architecture for implementing the skewed pixel processing order is presented in Section 5.4.

### 5.3. Encoder Front End

**Figure 19:**
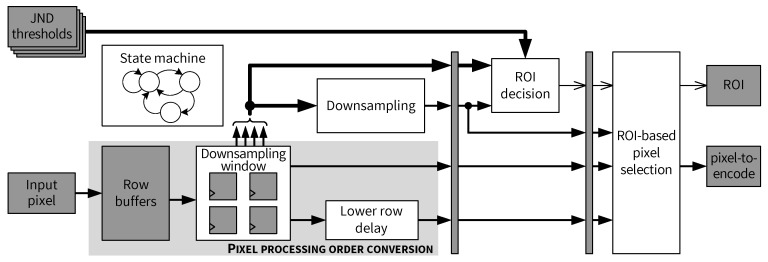
Encoder front end module.

A high-level architecture for the *Encoder front end* is presented in Figure 19. Input pixel buffering and skewed pixel output are performed in the *Pixel processing order conversion* module, which is composed mainly of shift registers and FIFOs as row buffers. When enough pixels are buffered so that the skewed processing can be started, pixels from the same columns in a pair of rows (called an upper row and a lower row in this paper) are outputted by the row buffers. After a full 2×2 pixel block is stored in the *Downsampling window*, the mean value of the block is computed by the *Downsampling* module. A *Lower row delay* block is used to delay the output of pixels on the lower row, as required by the skewed scan order. Figure 19 shows that all four original pixels in the *Downsampling window* and the output of the *Downsampling* module are sent to the *ROI decision* module, as well as the JND thresholds. Depending on whether the current 2×2 block is an ROI, either an original pixel or the downsampled mean value is adaptively selected by the *ROI-based pixel selection* module and forwarded to the predictive coding path. Different components of the encoder front end are connected by pipeline registers and their operation is controlled by a state machine. More details and architectural aspects of this module are examined in the following subsections.

### 5.4. Pixel Processing Order Conversion

**Figure 20:**
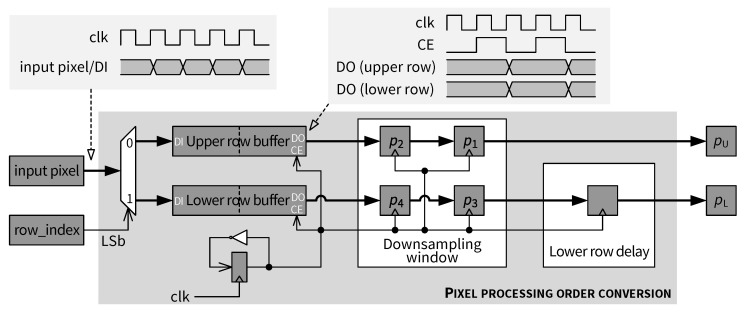
Pixel processing order conversion module.

The architecture of the *Pixel processing order conversion* module is shown in Figure 20. At the input side, pixels of the input image arrive sequentially (i.e., streaming scenario), as indicated in the waveform in the top-left side of Figure 20. According to the skewed scan order (cf. Figure 18), pixels in a pair of rows shall be interleaved with a delay in the lower row. As depicted in Figure 20, two different row buffers (dual-port RAMs) are used to store input pixels depending on the current row index. The modulo-2 operation on the row_index signal is implemented by taking the least significant bit (LSb) of row_index. The conversion process is as follows. Firstly, all pixels in an upper row (e.g., first row of the input image) are stored in the *Upper row buffer*. Next, pixels in a lower row (e.g., second row of the image) begin to be received and stored in the *Lower row buffer*. As long as neither row buffer is empty, both buffers are read simultaneously every two clock cycles, as illustrated in the waveform in the top-right side of Figure 20. Outputs of both row buffers are then fed into the *Downsampling window* consisting of two two-stage shift registers. Downsampling as well as ROI detection is performed once all 4 pixels of a 2×2 block are in the *Downsampling window*. Finally, by inserting an offset into the data path for the lower row pixels using the *Lower row delay* block, the skewed scan order [14] is obtained at the output of the *Pixel processing order conversion* module. The two output pixel values from the upper and lower rows are denoted as $p_{U}$ and $p_{L}$, respectively. Both $p_{U}$ and $p_{L}$ are candidates for the final pixel to be encoded, which is determined later by the *ROI-based pixel selection* module.

### 5.5. Downsampling and ROI Decision

**Figure 21:**
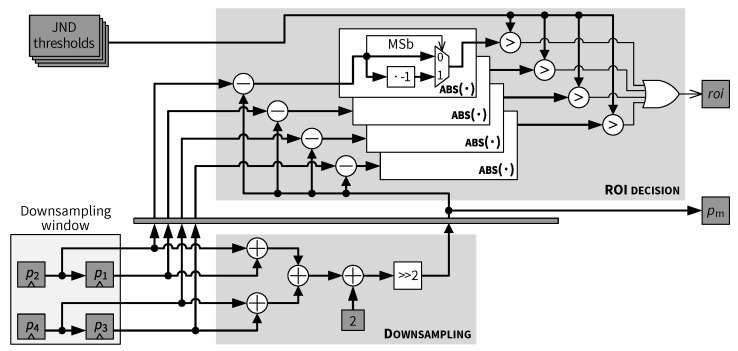
Downsampling and ROI decision modules.

The architecture of the *Downsampling* and *ROI decision* modules is presented in Figure 21. Let $p_{1},p_{2},p_{3},p_{4}$ be the four pixels of a 2×2 block in the downsampling window and $p_{m}$ be the downsampled mean value. The *Downsampling* module implements the following operation:

(29) $$p_{m}=\mathrm{ROUND}\left(\frac{p_{1}+p_{2}+p_{3}+p_{4}}{4}\right)$$

As shown in Figure 21, downsampling is performed by first adding up all 4 pixel values in an adder tree and then shifting right by 2 bits. The extra addition by 2 before the right shift is used to implement the rounding function in Equation (29). Such a downsampling scheme is straightforward and computationally efficient. When higher compression ratio is desired, the downsampling module and the corresponding register window and can be extended to deal with larger block sizes, and a low-pass filtering can be optionally employed before the downsampling to reduce aliasing.

The exploitation of the JND thresholds in the *ROI decision* module is illustrated in the upper part of Figure 21. The downsampled value $p_{m}$ is first subtracted from each of the original pixels $p_{1}$–$p_{4}$. The magnitude of a resulting difference value $|p_{i}-p_{m}|,i=\left\{1,2,3,4\right\}$ is the downsampling error at the *i*th pixel location in the current 2×2 block. Such a downsampling error is then compared with the corresponding difference visibility threshold $JND_{i}$. The current block is considered as an ROI (roi=1) if any downsampling error is greater than the corresponding JND threshold. Conversely, a non-ROI block (roi=0) is identified if all four downsampling errors are within the corresponding four JND thresholds. Downsampling can be applied to all non-ROI blocks without causing visual artifacts, since all pixels in a non-ROI block have visually “no difference” to the downsampled value of that block from a JND perspective.

### 5.6. ROI-Based Pixel Selection

**Figure 22:**
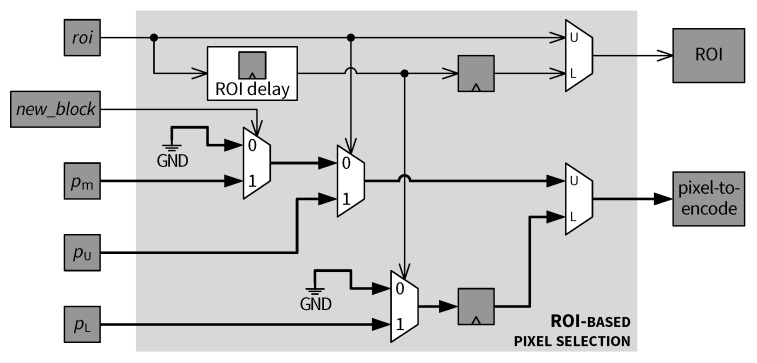
ROI-based pixel selection module.

The final pixels to be encoded are chosen by the *ROI-based pixel selection* module. Architecture of this module is depicted in Figure 22. The new_block signal is a binary control flag which is asserted when the upper row pixel register $p_{U}$ contains the first pixel of a new 2×2 block (see $p_{1}$ in Figure 16). Figure 19, Figure 20 and Figure 21 indicate that $p_{m}$, $p_{U}$ and roi signals are based on the same 2×2 block, i.e., these signals are synchronized with each other, whereas $p_{L}$ is delayed by one column compared with $p_{U}$. The *ROI delay* block generates an ROI status signal synchronized with $p_{L}$. The selection criteria are as follows.

(1) If the current 2×2 block is a non-ROI block (roi=0) and $p_{U}$ contains the first pixel of the block (new_block=1), then the downsampled pixel value $p_{m}$ is selected to replace $p_{U}$.
(2) If the current block is a non-ROI block (roi=0) and $p_{U}$ contains the second pixel of the block (see $p_{2}$ in Figure 16, new_block=0), then $p_{U}$ is skipped (i.e., pixel-to-encode is marked as invalid).
(3) A lower row pixel contained in $p_{L}$ is skipped if it is in a non-ROI block as indicated by the corresponding delayed ROI status signal.
(4) For any pixel, if the 2×2 block containing that pixel is an ROI block, then that pixel is selected for encoding, as shown in Figure 22.

Finally, the selected pixels, as well as the corresponding ROI flags, are transferred to the subsequent *Predictive coding* module, as indicated in Figure 17.

### 5.7. Predictive Coding and Output Bitstream

Pixels from the *Encoder front end* are compressed along the predictive coding path which comprises four main modules: *Prediction and context modeling*, *Symbol mapping*, *Coding parameter estimation* and *Golomb-Rice coding*, as depicted in the lower part of Figure 17. These blocks are implemented in a high throughput and resource efficient architecture for the classic context-based pixel-domain predictive coding, which is fully pipelined without stall. The throughput is 1 pixel/clock cycle. Architectural details here are similar to those in existing publications, e.g., on the hardware architecture for the regular mode of JPEG-LS [26]. The variable-length codeword streams from the predictive coding path are combined with the ROI (in raw binary representation) at the output multiplexing (*MUX*) module, where a barrel shifter is used to formulate fixed-length final output bitstreams. Detailed architecture for the predictive coding path and bitstream multiplexing is omitted due to space limitations.

## 6. Experimental Results

### 6.1. Analysis of Integer Approximation of the Gaussian Kernel

**Figure 23:**
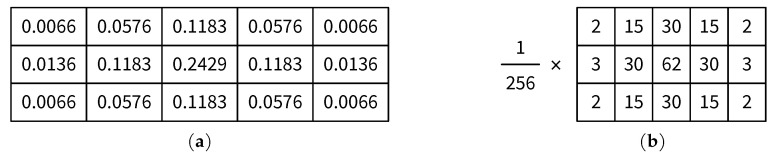
Coefficients of 3 × 5 Gaussian kernel in Section 3.1: (**a**) original; and (**b**) integer approximation.

As discussed in Section 3.1, a 3 × 5 Gaussian kernel with standard deviation σ=0.83 is employed in the proposed JND model. Figure 23a shows the original kernel coefficients with a precision of four digits after the decimal point, whereas an integer approximation of the same kernel is presented in Figure 23b. In total, 15 multiplications and 14 additions are required in a straightforward implementation of the filtering with the original kernel, whereas the integer kernel can be implemented with 25 integer additions plus several shift operations (for instance, multiplying *x* by 15 can be implemented by a shift-add operation as (x<<4)−x, where << is the left shift operator). The impact of using the integer kernel on the accuracy of results is analyzed in Table 1. The results using the integer kernel after both Gaussian smoothing and Sobel edge detection (cf. Figure 5) have been compared with those using the original kernel for various test images (see Section 6.2). Table 1 indicates that on average 97% of the results based on the integer version of the kernel matches those of the floating-point version after the smoothing step, whereas over 99% of the results based on the integer version of the kernel are the same as those based on the floating-point version after the edge detection step. Since the performance of the integer Gaussian kernel is closely comparable to that of the floating-point one, it is reasonable to use the integer kernel for the improved resource efficiency.

### 6.2. Performance of the Proposed JND Model

The proposed JND model was implemented in software and experimented with widely used standard test images. The performance of the proposed JND model was tested in terms of both the distortion visibility of JND-contaminated images and the amount of imperceptible noise that can be shaped into the images, i.e., visual redundancies in the images. To reveal or compare visual redundancies given by the JND models, the well-known PSNR metric is often used with a particular interpretation in the literature on JND models. For example, it is pointed out in [9] that, if the JND profile is accurate, the perceptual quality of the corresponding JND-contaminated image should be “as good as the original” while the PSNR of the JND-contaminated image should be “as low as possible”. Chou and Li believed that PSNR can be used to quantify the amount of imperceptible distortion allowed for transparent coding of images [9]. With this interpretation, a lower PSNR value corresponds to a larger potential coding gain. Other examples of work in which the PSNR metric is used in a similar way to analyze the performance of JND models include [11,12,27,28].

**Figure 24:**
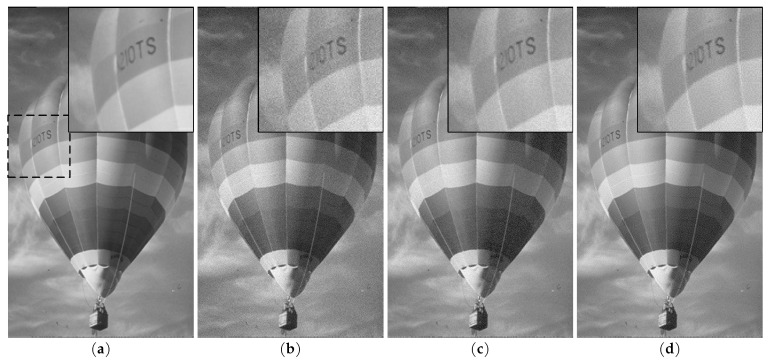
Visualization of JND-contaminated images: (**a**) original section of the BALLOON image; (**b**) contaminated with white noise, PSNR = 31.98; (**c**) contaminated with JND profile given by Chou and Li’s model [9] with random-sign injection, PSNR = 31.97; and (**d**) contaminated with JND profile given by the proposed JND model with zero-mean random-sign injection, PSNR = 31.74.

Multiple greyscale 8 bit/pixel test images [29,30] of different sizes and contents were used in our experiments. For each test image, four sets of JND profiles were computed using Chou and Li’s original model [9], Yang et al.’s model [11,22], Liu et al.’s model [12,31] and the proposed one. A JND-contaminated image was then obtained by injecting the JND profile as a noise signal to the original image. As described in [9], noise injection works by adding each original pixel with the corresponding visibility threshold multiplied by a random sign {−1,1}. The resulting JND-contaminated image can be used in both objective tests such as PSNR measurement to reveal the JND model’s capability for estimating the visual redundancy and subjective tests to validate the model by comparing the original image with the JND-contaminated one. Since each sign is generated independently, the above random-sign noise injection scheme may occasionally cause most injected noise samples in a small neighborhood to have the same sign, which often shows a correlation to distortion visibility even when the noise injection is guided by a high quality JND profile (see [13] for an example). An alternative is to ensure additionally a zero-mean of the randomly-generated signs of noise samples in every M×N block, which is referred to as zero-mean random-sign noise injection in this work. A neighborhood size of 2×2 in the zero-mean random-sign scheme was used in our experiments. The distortion visibility experiment on the proposed JND model was conducted on a $31.1^{\prime }$${}^{\prime }$ EIZO CG318-4K monitor with 100 cd/$m^{2}$ luminance and with viewing conditions specified in [32]. The original test image is temporal-interleaved with the JND-contaminated image at a frequency of 5 Hz, and a noise signal is invisible if no flickering can be seen. In our experiments, hardly any flickering could be noticed at a normal viewing distance corresponding to 60 pixels/degree. Figure 24 presents a test image and various noise-contaminated images. An original section of the BALLOON image is in Figure 24a, and a white-Gaussian-noise-contaminated image (PSNR = 31.98) is shown in Figure 24b. A JND-contaminated image (PSNR = 31.97) based on Chou and Li’s JND model is in Figure 24c, whereas the JND-contaminated image based on the proposed model is in Figure 24d. While the noise in Figure 24b is quite obvious, the same amount of noise injected based on Chou and Li’s JND model is much less visible (see Figure 24c), and an even higher amount (0.23 dB more) of noise based on the proposed model and the zero-mean random-sign injection scheme is almost completely invisible, as shown in Figure 24d.

Table 2 shows a comparison of PSNR values of JND-contaminated images based on different JND models. As discussed above, the PSNR metric was used as an indication of visual redundancy measured by a JND model, which can be removed without impairing the visual quality. A lower PSNR value is preferable since it corresponds to a more accurate estimation of the visual redundancy, which can be used to guide a visually lossless image coding or watermarking. Table 2 indicates that the proposed JND model on average improved the accuracy of visual redundancy estimation by 0.69 dB and 0.47 dB compared to Chou and Li’s model and Yang et al.’s model, respectively. Compared with Liu et al.’s model, which applies on top of Yang et al.’s model an additional total-variation-based textural image decomposition [12], the average accuracy of the proposed model was lower by 0.6 dB. Such a gap could be justified by the relatively low computational complexity of the proposed model, especially for resource-constrained embedded systems.

### 6.3. Complexity Comparison of Proposed JND Model and Existing JND Models

Table 3 lists the number of operations required by Chou and Li’s JND model, which is the basis for the other pixel-domain JND models discussed in this paper. The complexity of two JND models extending Chou and Li’s model, including Yang et al.’s model and the proposed one, are compared in Table 4 in terms of the number additional operations required in the main algorithmic parts of these JND models. Compared with Chou and Li’s JND model, Yang et al.’s model additionally performs edge-based weighting of the contrast masking effect using a Canny edge detector followed by a 7×7 Gaussian filter [9]. From the upper part of Table 4, it can be seen that Yang et al.’s model required approximately 162 additions, one multiplications, one division and a look-up table (LUT) in addition to the basic operations required in Chou and Li’s model (Table 3). It can be seen from the lower part of Table 4 that compared to Yang et al.’s model, the proposed model required about half the number of extra additions and required neither additional LUTs nor division operations.

A comparison of software complexity in terms of CPU time was made for different JND models. The comparison was based on the original authors’ implementation of Yang et al.’s model [22] and Liu et al.’s model [31], as well as our own implementation of Chou and Li’s model and the proposed one. All models were implemented in MATLAB. The software models were run on a desktop computer with Intel Core i7-4820K (3.70 GHz) CPU and 32 GB of RAM. The operating system was Windows 7 64-bit. The test image used was BARB with a resolution of 720 × 576. The time need by each model to evaluate the JND profile was obtained as the least CPU time measured from running each JND model 30 times on the test image. The results are presented in Table 5. It can be seen that the CPU time required by the proposed model to evaluate the JND profile was 68 ms, which was less than twice of that (37 ms) required by Chou and Li’s model. By contrast, the CPU time required by Yang et al.’s model was 88 ms, which was more than twice of that required by Chou and Li’s model. In the case of Liu et al.’s model, the CPU time was 474 ms, which was over an order of magnitude more than that of Chou and Li’s model.

To compare the JND models in terms of hardware resource requirement and speed, we implemented hardware models of three JND models in VHDL, including Chou and Li’s original model, Yang et al.’s model and the proposed one. The hardware models were simulated and synthesized using Xilinx Vivado Design Suite 2018.2. The target device was selected as Xilinx Kintex-7 XC7K160T with a speed grade of −2. For the FPGA implementation of the proposed JND model, the input image was assumed to be greyscale with 8 bits/pixel and with a horizontal size of up to 1024 pixels. Table 6 presents the FPGA resource utilization of the synthesized models and their maximum clock frequency. The pixel throughput was one pixel per clock cycle. Table 6 shows that, compared with Chou and Li’s JND model, the amount of required FPGA hardware resource was increased by over 200% for Yang et al.’s JND model, while for the proposed model the resource increase was less than 100%. In terms of the maximum clock frequency, the proposed model achieved the same performance as Chou and Li’s model, i.e., 190 MHz, which was about 35% faster than the 140 MHz achieved by Yang et al.’s model.

### 6.4. Compression Performance of the Perceptual Codec Based on the Proposed JND Model

The proposed JND model was implemented in combination with the perceptual encoder described in Section 5. Parameter values for the JND model are as discussed in Section 3. Compressed image quality of the perceptual codec was compared with that of JPEG-LS for a range of rates corresponding to approximately 2:1 to 6:1 compression. Objective metrics used to evaluate the compressed image quality included PSNR, MS-SSIM [36,37] and HDR-VDP score [38,39]. Compressed data rates of the perceptual codec based on the proposed JND model were additionally compared with those of JPEG, JPEG 2000 and JPEG XR at the same perceptual quality given by PSPNR [9]. The compression experiments were based on widely used standard test images, as described in Section 6.2.

**Figure 25:**
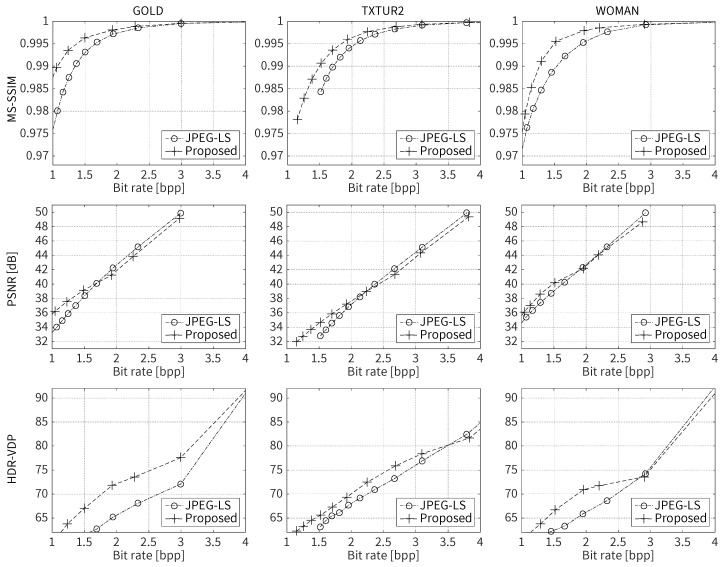
Objective rate-distortion plots of the proposed codec and JPEG-LS: top to bottom, MS-SSIM, PSNR and HDR-VDP values; and left to right, results for test images GOLD, TXTUR2 and WOMAN.

**Figure 26:**
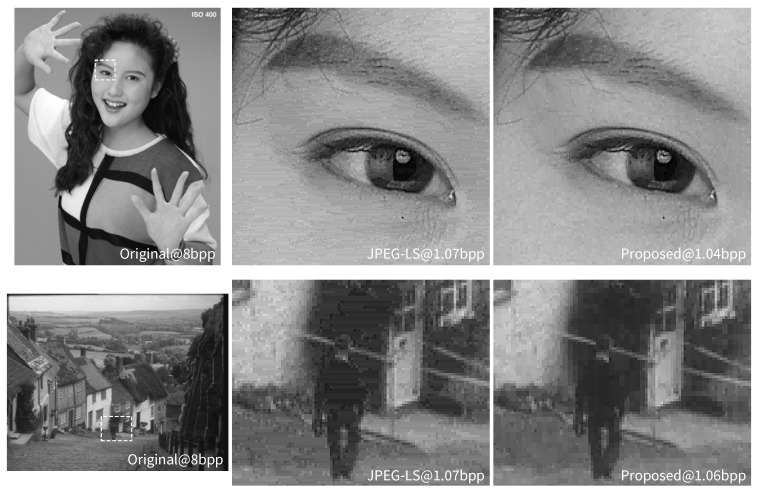
Visual quality of images compressed by JPEG-LS and the proposed JND-based perceptual codec at closely comparable bit rates: top and bottom, WOMAN and GOLD image; and left to right, original image, selected section compressed by JPEG-LS, and same section compressed by the perceptual codec.

Figure 25 presents comparisons of rate-distortion performance between the perceptual codec based on the proposed JND model and JPEG-LS for test image GOLD, TXTUR2 and WOMAN. It can be seen from the MS-SSIM and HDR-VDP curves that the perceptual codec exhibited a clear gain in perceptual quality over JPEG-LS in a rate range between 1 and 3.5 bits-per-pixel (bpp). In terms of PSNR, which is not a perceptual quality metric, the perceptual codec delivered an improved coding performance of about 10–15% over JPEG-LS at rates below approximately 1.5–2 bpp. Figure 26 provides visual comparisons of images compressed to approximately the same rate by JPEG-LS and the perceptual codec combined with the proposed JND model. Selected parts of two different types of images are shown. From this figure, it is evident that the proposed scheme achieved improved visual quality by avoiding the stripe-like artifacts of JPEG-LS.

Towards the goal of visually transparent coding, a codec’s performance can be related to its ability to keep coding distortions within the visibility thresholds provided by the JND model. As discussed in [9], the peak signal-to-perceptible-noise ratio (PSPNR) is a metric taking visual redundancy into account based on the JND model.

While transform-domain codecs such as JPEG, JPEG 2000 and JPEG XR have higher complexity and latency than a pixel-domain codec such as the proposed JND-based one or JPEG-LS, it is possible to find out experimentally the bit rates at which any coding distortion in the compressed image is kept below the corresponding visibility threshold given by the proposed JND model. Table 7 shows the minimum compressed bit rates for JPEG, JPEG 2000, JPEG XR and the proposed JND-based perceptual codec at which the PSPNR reaches the upper bound, i.e., none of the coding errors exceed the JND thresholds, which can be considered as a necessary condition given by the JND model on perceptually lossless coding. For this experiment, the proposed JND model, the baseline JPEG, Kakadu implementation [40] of JPEG 2000 (with visual weights) and the ITU-T reference implementation [41] of JPEG XR were used. Table 7 indicates that, at the same visual quality given by PSPNR, the perceptual codec required on average about 58%, 48% and 41% fewer bits compared with JPEG, JPEG 2000 and JPEG XR, respectively.

### 6.5. FPGA Resource Utilization and Throughput of the Proposed Perceptual Encoder Architecture

The architecture for the proposed JND model and perceptual encoder was implemented in hardware using VHDL hardware description language. The hardware model for the perceptual encoder was simulated and synthesized using Xilinx Vivado Design Suite 2016.4. The target device was selected as Xilinx Kintex-7 XC7K160T, a popular mid-range FPGA, with a speed grade of −2. Since the proposed perceptual encoder is compatible with different JND models (and vice-versa for the proposed JND model), the proposed JND model and perceptual encoder were implemented as separate modules, and their synthesis results are reported separately for clarity. An integration of these two modules is straightforward, as is obvious from Section 5. Synthesis results for the proposed JND model as well as two other JND models are presented in Section 6.3.

Table 8 shows the FPGA resource utilization of the proposed perceptual encoder architecture for 8–16 bits/pixel input greyscale images with a horizontal size of up to 2048 pixels. It can be seen that the proposed encoder architecture required 5.85% of logic resource and 2% of the BRAM resource on the target FPGA, and a pixel throughput of about 140 Megapixel/s (1 pixel/clock cycle) was achieved. For both the proposed JND model and the perceptual encoder architecture, the logic and BRAM resources used were well below 10% of all the available resources of each type on the target FPGA, which, on the one hand, provides abundant hardware resources for the other image processing tasks running on the FPGA such as noise cancellation, and, on the other hand, leaves ample room for using multiple parallel encoding instances on a single FPGA when higher pixel throughput is demanded.

## 7. Conclusions

A new pixel-domain JND model and a perceptual image coding architecture exploiting the JND model are presented. In the proposed JND model, lightweight and hardware-efficient operators are used to identify edge, texture and smooth regions in the input image. Different weighting factors for the contrast masking effects are applied to pixels in different regions. The contrast masking and luminance masking effects are combined into the final JND value in the new approach, i.e., using the nonlinear additivity model for masking (NAMM) operator for texture/smooth regions and the maximum operator for edge regions. The proposed JND model and architecture are suitable for implementation on FPGAs for real-time and low complexity embedded systems. In the proposed architecture for a low complexity pixel-domain perceptual codec, the input image is adaptively downsampled based on the visual ROI map identified by measuring the downsampling distortion against the JND thresholds. The proposed JND model provides a more accurate estimation of visual redundancies compared with Chou and Li’s model and Yang et al.’s model. Since the computational complexity of the proposed model is significantly less than that of Liu et al.’s model based on image decomposition with total variation, the proposed JND mode achieves a new balance between the accuracy of JND profile and the computational complexity. Experimental results further show that the proposed JND-based pixel-domain perceptual coder achieved improved rate-distortion performance as well as visual quality compared with JPEG-LS. At the same perceptual quality in terms of PSPNR, the proposed coder generated fewer bits compared with JPEG, JPEG 2000 and JPEG XR. Finally, FPGA synthesis results indicate that both the proposed JND model and the perceptual coder required a very moderate amount of hardware resources to implement in terms of both logic and block memory resources. On a mid-range FPGA, the hardware architecture of the proposed JND model required about 2.6% of logic and 1.4% of block memory resources and achieved a throughput of 190 Megapixel/s, while the hardware architecture of the perceptual encoder required about 6% of logic and 2% of block memory resources and achieved a throughput of 140 Megapixel/s.

## Figures and Tables

**Table 1:** Influence of the integer Gaussian kernel on the accuracy of smoothing and edge detection results in comparison with the original kernel in floating-point double precision.

Average Ratio of Pixel Locations with Same Results Using the Integer Kernel and the Original One	
**After Gaussian Smoothing**	**After Sobel Edge Detection**
97.00%	99.89%

**Table 2:** Performance comparison of different JND models for measuring the visual redundancy in test images based on PSNR values of JND-contaminated images.

Image	PSNR [dB]			
	Chou & Li [9]	Yang et al. [11]	Proposed	Liu et al. [12]
AERIAL2	33.11	32.23	32.01	31.52
BALLOON	31.97	31.89	31.74	31.57
CHART	30.91	31.92	30.65	30.35
FINGER	32.69	33.49	31.50	29.24
GOLD	30.93	30.32	30.18	29.81
HOTEL	29.92	29.96	29.44	28.85
MAT	32.22	32.40	31.87	31.46
SEISMIC	37.84	36.35	36.83	36.46
TXTUR2	32.06	31.05	30.60	30.04
WATER	34.18	34.44	34.06	34.01
WOMAN	30.94	30.22	30.22	29.25
Average	32.43	32.21	31.74	31.14
Improvement vs. Chou & Li	–	0.22	0.69	1.29

**Table 3:** Basic operations required for computing a visibility threshold by Chou and Li’s JND model.

Algorithmic Step	Addition	Multiplication	LUT	Remark
BL	24	–	–	Equation (1)
ID	44	–	–	Equation (3)
MG	3	–	–	Equation (4)
α	1	1	–	Equation (6)
β	1	1	–	Equation (7)
final CM	1	1	–	Equation (5)
LM (BL≤127)	–	–	1	Equation (2)
LM (BL>127)	3	–	–	Equation (2)
Final JND	1	–	–	Equation (8)
Total	78	3	1	

**Table 4:** Approximate number of additional operations per pixel required for computing a visibility threshold by Yang et al.’s JND model and the proposed model.

Model	Algorithmic Step	Addition	Multiply	LUT	Division	Remark
Yang’s C: Canny	C: smoothing	37	–	–	1	σ=1.4 [33]
	C: gradients	10	–	–	–	Sobel
	C: gradient-magnitude	1	–	–	–	[24]
	C: gradient-direction	3	–	1	–	[24]
	C: non-max suppression	2	–	–	–	[24]
	C: gradient-histogram	2	–	–	–	[34]
	C: 2-thresholding & hysteresis	2	–	–	–	[35]
	7×7 Gaussian	102	–	–	–	σ=0.8 [11]
	Edge-weighting	–	1	–	–	[11]
	NAMM	3	1	–	–	Equation (12)
	Total	162	2	1	1	
Proposed E: edge T: texture	E: 3×5 smoothing	25	–	–	–	Figure 23b
	E: Sobel gradients	10	–	–	–	Equation (26)
	E: magnitude	1	–	–	–	Figure 13b
	E: thresholding	1	–	–	–	Figure 13b
	T: local contrast	26	–	–	–	Equation (27)
	T: contrast significance	1	–	–	–	Equation (14)
	T: contrast activity	8	–	–	–	Equation (15)
	T: high activity	1	–	–	–	Equation (16)
	$CM_{t}$ weighting	1	–	–	–	$W_{t}\;=\;1.75$
	Final JND	6	2	–	–	Equation (20)
	Total	80	2	–	–	

**Table 5:** CPU time used by MATLAB implementations of different JND models for evaluating the JND profile of the BARB test image.

	Chou & Li	Yang et al.	Liu et al.	Proposed
CPU time (ms):	37	88	474	68
Increase vs. Chou & Li:	–	138%	1181%	84%

**Table 6:** FPGA resource utilization and clock frequency comparison of three JND models: Chou and Li’s model, Yang et al.’s model and the proposed one.

Resource Type	Available	Chou & Li	Yang et al.	Proposed
Slice LUTs	101,400	1414 (1.39%)	4128 (4.07%)	2621 (2.58%)
Slice Registers	202,800	839 (0.41%)	2482 (1.22%)	1543 (0.76%)
Block RAM 36Kbits	325	2.5 (0.77%)	10.5 (3.23%)	4.5 (1.38%)
Clock frequency (MHz)		190	140	190

**Table 7:** Compressed data rates of JPEG, JPEG 2000, JPEG XR and the proposed JND-based perceptual encoder at the same quality in terms of peak signal-to-perceptible-noise ratio (PSPNR).

Image	Bit Rate (bpp)			
	JPEG	JPEG 2000	JPEG XR	Proposed
AERIAL2	6.04	5.10	4.44	2.68
BABOON	7.03	5.50	4.91	3.37
BALLOON	2.60	2.19	1.58	0.97
BARB	4.37	3.89	3.31	2.14
BOATS	4.11	3.70	3.19	1.75
CAFE	6.29	4.81	4.51	2.54
CATS	2.88	2.20	2.06	1.45
CHART	3.58	2.80	2.53	1.37
EDUC	4.50	3.96	3.53	2.21
FINGER	5.91	4.70	4.40	3.01
GOLD	5.00	4.00	3.42	1.93
HOTEL	4.98	3.90	3.46	1.74
LENNAGREY	4.64	3.70	3.34	1.69
MAT	3.61	2.50	2.44	1.23
PEPPERS	4.93	4.10	3.54	1.85
SEISMIC	2.11	1.88	1.46	1.30
TOOLS	6.26	5.09	4.58	2.68
TXTUR2	6.31	5.20	4.47	2.68
WATER	3.55	2.89	2.55	1.03
WOMAN	5.01	4.19	3.56	1.96
Average	4.69	3.82	3.36	1.98
Saving by perceptual encoder	57.8%	48.1%	41.2%	–

**Table 8:** FPGA resource utilization of the proposed perceptual encoder architecture.

Resource Type	Used	Available	Percentage
Slice LUTs	5934	101,400	5.85%
Slice Registers	2300	202,800	1.13%
Block RAM 36Kbits	6.5	325	2%
Clock frequency (MHz)	140		
